# Enzymatic Analysis of Iranian *Echis carinatus* Venom Using Zymography

**Published:** 2017

**Authors:** Mostafa Kamyab, Euikyung Kim, Seyed Mehdi Hoseiny, Ramin Seyedian

**Affiliations:** a *Faculty of Biological Sciences, Shahid Beheshti University, Tehran, Iran. *; b *Department of Pharmacology and Toxicology, College of Veterinary Medicine, Gyeongsang National University, Jinju, South Korea. *; c *Department of Pharmacology and Toxicology, Bushehr University of Medical Sciences, Bushehr, Iran.*

**Keywords:** *Echis carinatus*, Zymography, Antivenom, Gelatinase

## Abstract

Snakebite is a common problem especially in tropical areas all over the world including Iran. *Echis carinatus* as one of the most dangerous Iranian snakes is spreading in this country excluding central and northwest provinces. In this study gelatinase and fibrinogenolytic properties as two disintegrating matrix metalloproteinase enzymes were evaluated by a strong clear halo between 56-72 kDa in addition to another band located 76-102 kDa for gelatinase and one major band around 38 kDa for fibrinogenolytic enzyme respectively. The electrophorectc profile of our venom demonstrated at least one protein band between 24-31 kDa like previous reports and another two bands between 52-76 kDa and below 17 kDa stemmed probably due to the effect of natural selection in one species. According to our results Razi institute antivenin could neutralize *in-vitro* effects of gelatinase enzyme comprehensively. The electrophoretic profile of Iranian commercial antivenom as the main intravenous treatment of envenomed patients showed impurities in addition to F (abʹ)^2^ weighing 96 kDa in SDS-PAGE analysis. It proposes more efforts for refinement to avoid short and long unwanted effects in envenomed patients.

## Introduction

Snake bite is a major cause of life-threatening conditions including terror, nausea, vomiting, syncope and tachycardia in human. Contrary to public opinion, only 15 percent of the approximately 3000 species of these ceatures are dangerous to humans (1) leading to 25,000-125,000 deaths annually worldwide (2). Based on available facts, snake bite is an emergency and dangerous to human beings especially in tropical and subtropical areas like India and Iran (3, 4). *Echis* *Carinatus* (saw-scaled viper) is one of the most venomous snakes found in Africa and Middle east (5) causing hemorrhage, oliguria, anuria and in the most severe cases acute renal failure due to disseminated intravascular coagulation (6) in envenomed patients. Local and systemic bleeding induced by envenomation with this snake bite is directly related to metalloenzymes like gelatinase and sphingomyelinase in the vasculature (7). Antivenom injection obtained by immunization of horses is the recommended therapy for envenomed patients, but in severe cases factor replacement therapy could be used. According to few studies that have been done on the evaluation of Iranian *Echis carinatus *venom and its pathological enzymes, the purpose of our study was *in-vitro* investigation of gelatinase enzyme as one of the most potent metalloenzymes and the fibrinogenolytic property. *In-vitro *neutralizing role of Razi Institute antivenom was assessed via zymographic method and its purity for avoiding unwanted effects in envenomed patients was evaluated.

## Experimental

*Reagents*

Gelatin from porcine skin, fibrinogen from human plasma and molecular weight size marker for electrophoresis were purchased from Sigma Aldrich Co (St Louis Co, USA). All other reagents were analytical grades from commercial sources.

**Figure 1:**
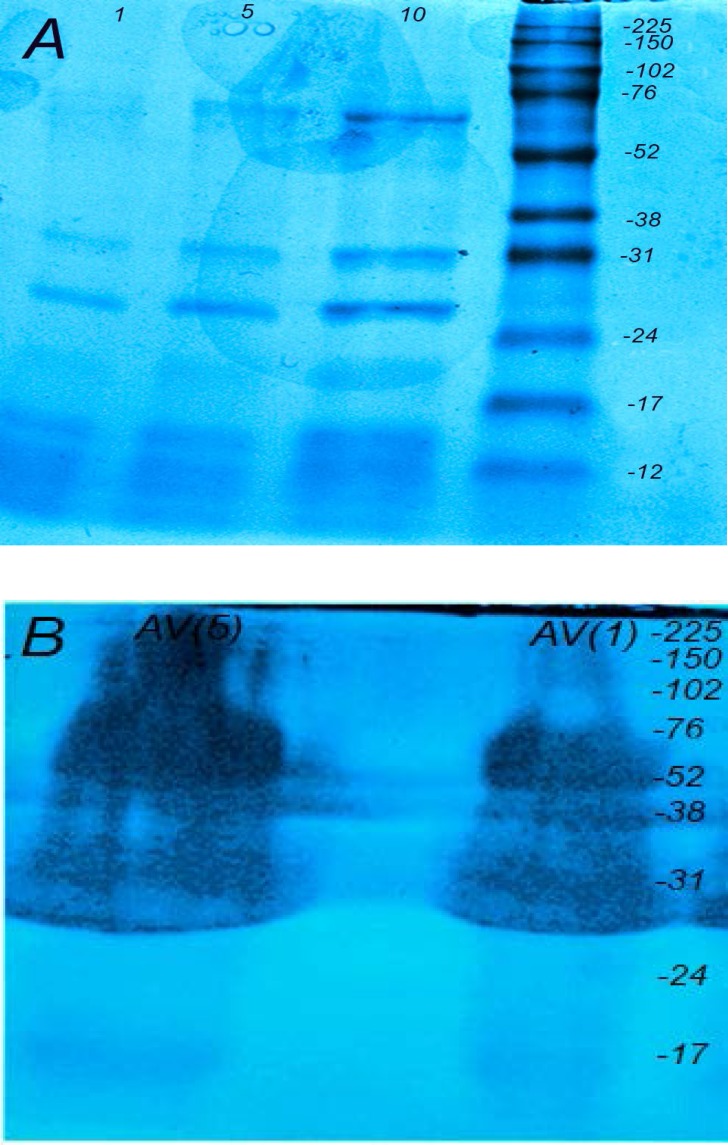
Protein components of* Echis carinatus* venom and Razi Institute antivenom. (A) The protein ingredients of *Vipera.lebetina* venom were separated with SDS-PAGE (12.5%) and stained with coomassie blue dye. (LANE 1:1, lane 2:5 and lane 3:10 μg). (B) Razi Institute antisnake antivenin was run on a 12.5% SDS-PAGE and stained with coomassie blue dye (lane 1:5 and lane 2:1 μg). Numbers on the right indicate the molecular weight of size markers

**Figure 2:**
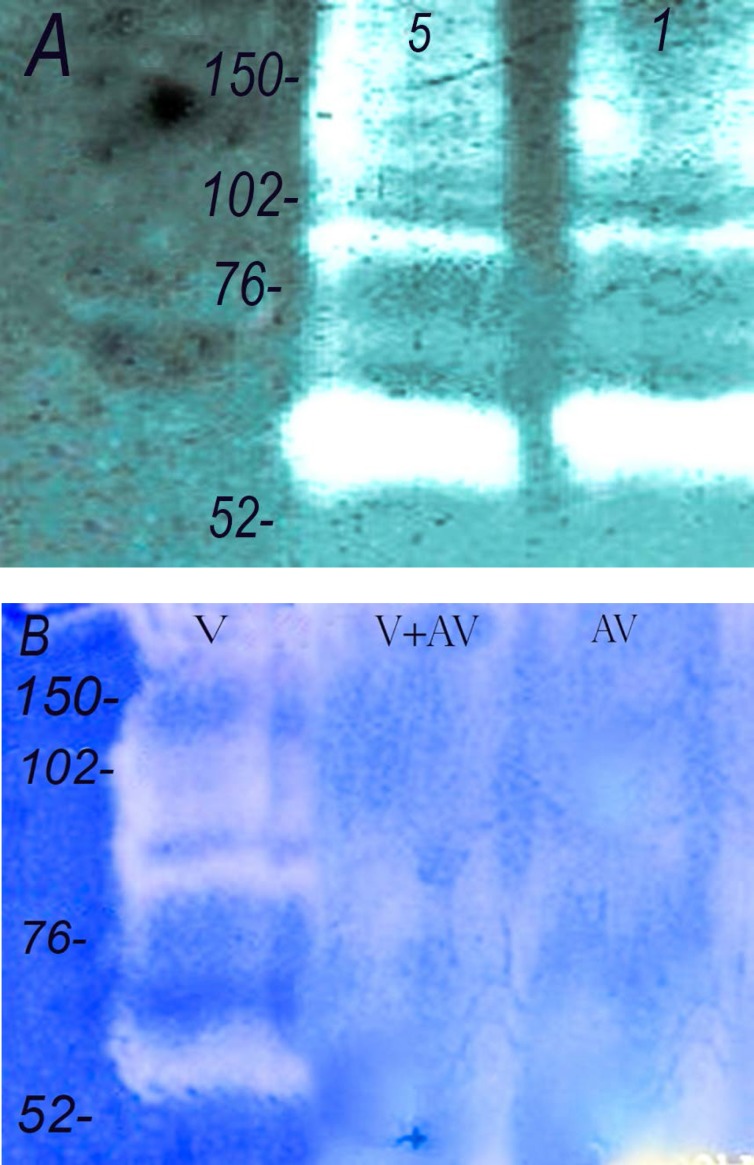
Gelatinase activity of *Echis carinatus *venom. Zymographic studies were performed for gelatinase activity. (A) The venom (lane 1 : 5 and lane 2 : 1 µg) was run on 12.5% containing gelatin as described in methods. (B) Neuralization of the gelatinase activity. (lane 1: venom alone, 1 µg, lane 2: venom 1 µg + antivenom, 5µL and lane 3: antivenom, 5 µL). Numbers on the left indicate the molecular weight of size markers

**Figure 3:**
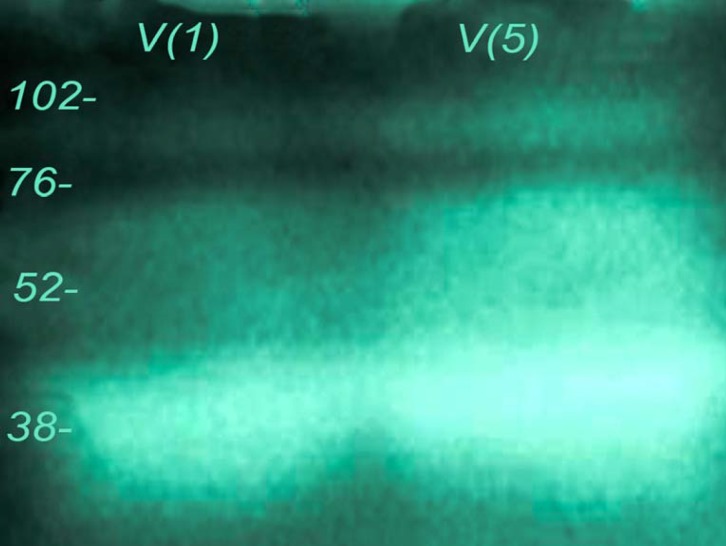
Fibrinogenolytic activity of *Echis carinatus* venom. Zymographic experiment was performed to investigate this property. The venom (lane 1 :1 and lane 2 : 5 µg) was run on 12.5% containing fibrinogen as described before

*Venom and polyvalent antivenom preparation*

Venoms were obtained from snakes collected in different parts of Iran and kept at the serpantarium of the Razi Institute of Vaccine and Serum production. Once milked, it was stored at -20 °C and freeze dried until reconstitution in our laboratory by normal saline. The antivenom used was the polyvalent against 6 different venoms (*Naja naja oxiana,Vipera lebetina, Echis carinatus, Vipera albicurnata, Agkistrodn halys *and *Pseudoceratus*) by immunization of horses. The protein concentrations of the venom and polyvalent antivenom were determined by Bradford method (8).

*SDS-PAGE*

The protein components of *Echis carinatus* venom (5, 10 and 15µg) and Razi Institute polyvalent antivenom (1 and 5 µg) were determined using SDS-PAGE 12.5% acryl amide gels under non reducing condition separated by the method of Laemlli (9). Gels were stained with Coomassie blue R-250 after electrophoresis for determination of protein bands. Molecular mass markers were included in all runs.

*Gelatinase and Fibrinogenolytic assay*

In order to study the gelatinase or fibrinogenolytic activity of this venom and neutralization property of Razi Institute antivenom, SDS-PAGE (12.5%) was prepared and polymerized with gelatin or fibrinogen (1%) for our experiment (10, 11). Electrophoresis was carried out using 15 mA in cold temperature. The gels were washed in Triton X-100 for 30 min and rinsed with purified water to remove SDS and incubated overnight at 37 °C in reaction buffer (Tris base: 1.2 g, Tris-HCl: 6.3 g, NaCl: 11.7 g, CaCl2: 0.74 g) dissolved in 1 liter of distilled water (12).

After incubation, the gels were stained with Coomassie blue solution and destained for 30 minutes in 7.5% acetic acid and 5% methanol. The clear zone of substrate on blue background gels indicated the presence of gelatin and fibrinogen degrading activities dependent on the substrate used in the assay (13).

## Results

*SDS-PAGE analysis*

The electrophoretic profiles of *Echis carinatus *venom with 12.5% acrylamide demonstrated at least three major protein bands below 17, 24-31 and 52-76 kDa (Figure 1A). Moreover, Razi Institute snake antivenom was examined by 12.5% acrylamide for characterizing its protein components (Figure 1B). The protein bands could be detected between 31-150 kDA.

*Gelatinase and fibrinogenolytic results*

*Echis carinatus* venom showed significant amounts of enzyme activities against gelatin and fibrinogen in different experiments (Figures 2 and 3). In our zymographic technique for finding gelatinase property, we found one major band located between 52-76 kDa and another minor band at 76-102 kDa. Gelatinase activity was completely neutralized by incubation of venom and Razi institute antivenom as displayed in Figure 2B. Antivenom had no gelatinase activity in our experiment. *Echis carinatus *venom had a strong fibrinogenolytic activity at 38 kDa in our zymographic experiment with clear band on background gel (Figure 3).

## Discussion

More than one hundred thousand species among all major phyla are known as venomous creatures that widely distributed in the world. Venom as a deadly cocktail of bioactive component is one of the most exciting technique of snake for capturing prey or defense (14). Determination of the actual annual amount of snakebites and its death tolls in the world is impossible due to lack of statistics from some countries (15). The last discontinuous estimates reveals that venomous snakes cause around 5.4 million bites, about 2.5 million envenoming and over 125,000 death annually (16, 17). There are 83 species of snakes in Iran, including nonvenomous (forty-five snakes), venomous (twenty-seven snakes) and semivenomous (eleven snakes) in addition to five sea snakes (eighteen snakes). Snakebite is one of the most important health issues especially in tropical areas like Middle East. Intravenous administration of Razi Institute antivenom in addition to reassuring and calming the patient is usually applied for envenomed patients. *Echis carinatus* (the capet or saw scaled viper) belongs to viperidea famioy as one of the most dangerous snakes is distributed in Iran excluding central and north west provinces (19). This venom which contains a cocktail of different fibrinigenolytic and hemorrhagic metalloproteinase enzymes causes mortality in humans more than other snakes in Iran (20). The electrophoretic profile of our venom revealed by SDS-PAGE (12.5%) showed one major band at 30 kDa similar to previous reports (21, 22) and three other bands below 17, 24-31 and 52-76 kDa (Figure 1A) that were not shown in Iranian records. Our SDS-PAGE result was comparatively similar to previous Pakistani report (23). It seems that natural selection in addition to time of venom collection have great roles in its composition in the same indivisual species (24, 25). Gelatinase and fibrinogenolytic properties related to matrix metalloproteinase family are known as deleterious enzymes in *Echis carinatus *leading to devastating effects in envenomed patients. According to Zymographic technique as a simple, favorable and reliable experiment, there was one major gelatinase band at 52-76 kDa and another minor componet at 76-102 kDa in addition to one major fibrinogenolytic band at 38 kDa similar to the previous reports showing the relevant proteins specially in viperidae family (26, 27). 

It is noteworthy that where availabe, intravenous infusion of snake antivenom is the best effective treatment against snakebite while standard treatment for coagulopathies induced by envenomation, such as factor replacement and heparin are ineffective (28). According to Figure 2B, it is presumed that the Iranian commercial antivenin produced by Razi Vaccine and serum research Institute neutralizes *in-vitro* *Echis carinatus* gelatinase activity as one of the greatest protease enzymes in a concentration-dependent manner similar to foreign antivenoms. In our study the purity of Iranian antivenom as a sterile preparation containing equine immunoglobulin fragments F(abʹ)2 was investigated. According to Figure 1B, the protein bands of polyvalent antivenin were distributed in a wide range from 31 kDa to 220 kDa by resolving with SDS-PAGE (12.5%). From our analysis, it seems that minority of this antivenin appears to be composed of F(abʹ)2 with molecular weight of 96 kDa possibly leading to adverse short and late reactions in envenomed patients due to type III hyperreactivity. At the end, zymography as a reliable and effective technique could be further used for detection of matrix metalloproteinase enzymes degrading fibronectin, laminin, collagen IV and other substances of *Echis carinatus* venom as one of the poisonous snakes of Iran (29). Our findings on Razi institute polyvalent antivenin demonstrates its *in-vitro* neutralizing properties on gelatinase activity but it is suggested that more efforts must be carried out on the refinement and concentration processes for preparation of higher qualities of this antivenin in order to avoid unwanted effects in envenomed patients. 
